# Influence of Dzyaloshinskii–Moriya interaction and perpendicular anisotropy on spin waves propagation in stripe domain patterns and spin spirals

**DOI:** 10.1038/s41598-023-28271-2

**Published:** 2023-01-21

**Authors:** Pawel Gruszecki, Jan Kisielewski

**Affiliations:** 1 Institute of Spintronics and Quantum Information, Faculty of Physics, Adam Mickiewicz University, Poznań, 61-712 Poland; 2 Faculty of Physics, University of Białystok, Białystok, 15-245 Poland

**Keywords:** Ferromagnetism, Spintronics

## Abstract

Texture-based magnonics focuses on the utilization of spin waves in magnetization textures to process information. Using micromagnetic simulations, we study how (1) the dynamic magnetic susceptibility, (2) dispersion relations, and (3) the equilibrium magnetic configurations in periodic magnetization textures in a ultrathin ferromagnetic film in remanence depend on the values of the Dzyaloshinskii–Moriya interaction and the perpendicular magnetocrystalline anisotropy. We observe that for large Dzyaloshinskii–Moriya interaction values, spin spirals with periods of tens of nanometers are the preferred state; for small Dzyaloshinskii–Moriya interaction values and large anisotropies, stripe domain patterns with over a thousand times larger period are preferable. We observe and explain the selectivity of the excitation of resonant modes by a linearly polarized microwave field. We study the propagation of spin waves along and perpendicular to the direction of the periodicity. For propagation along the direction of the periodicity, we observe a bandgap that closes and reopens, which is accompanied by a swap in the order of the bands. For waves propagating in the perpendicular direction, some modes can be used for unidirectional channeling of spin waves. Overall, our findings are promising in sensing and signal processing applications and explain the fundamental properties of periodic magnetization textures.

## Introduction

Magnetic texture-based magnonics^1–3^ is a subfield of magnonics^4^ focused on spin wave (SW) dynamics in non-uniform magnetization textures, such as single domain wall^5–8^, skyrmion^9^, or periodic magnetization textures such as skyrmion lattices^10,11^ or stripe domain patterns^12–15^. The primary advantages of using magnetization textures as a medium for SW propagation with respect to uniformly magnetized nanofabricated films are (1) no need for complex nanostructuring and reduced impact of defects, (2) reprogrammability, and (3) the ability to couple magnetization dynamics with the magnetization textures. The latter one enables the emission of short-wavelength SWs^16–21^ and nonlinear interactions of SWs with the texture itself^22–25^. Magnetic domain walls can be used as ultra-narrow waveguides^7,8,26–28^, phase shifters^6,29–32^, or polarizers^33^. The SW dispersion relation in periodic magnetic textures is also periodic^34^ and may possess bandgaps^12,14,35^, therefore, periodic magnetization textures can serve as magnonic crystals^36,37^ with lattice constants down to tens of nanometers that are unattainable by other techniques^12,13,38^. These systems are also of great interest from a fundamental point of view, e.g., Goldstone and Higgs modes can be found there^39^.

Basic magnetic parameters determining the internal structure and chirality of magnetization textures are film’s thickness, quality factor (*Q*) being the ratio of the perpendicular magnetocrystalline anisotropy (PMA) to demagnetization energies, and the Dzyaloshinskii–Moriya interaction^40,41^ (DMI). Together with the exchange constant and the saturation magnetization, these parameters determine the periodicity of magnetization textures^38,42^. Besides, DMI also influences SW flow in uniformly magnetized thin films introducing nonreciprocity^43–46^.

Spin spirals are type of the fundamental magnetic configurations in thin films with DMI and PMA through which SWs can propagate^47,48^. Spin spirals, due to the chiral nature of DMI, are characterized by a continuous chiral rotation of magnetization in the plane parallel to the direction of periodicity. Intriguingly, the spatial distribution of spin spiral, in which magnetic moments rotate in a plane parallel to the direction of propagation, resembles the profile of Damon–Eshbach SWs in ultra-thin films. Although the spectrum of resonance modes in periodic stripe domain patterns has been studied for years^49–52^, spin spirals, through which SWs can also propagate, have not been extensively studied as one-dimensional magnonic crystals.

In this paper, we study how spin spirals’ and stripe domain patterns’ static and dynamic properties depend on DMI and PMA and show transition between the two states. We analyze the influence of DMI and PMA changes on the resonance spectrum. We focus on the selectivity of the excitation of resonances, analyze the propagation of SWs, show the prospects of using spin spirals as magnonic crystals, and reveal the fundamental properties of magnetization disturbances propagation in such systems. We conclude this paper by analyzing the propagation along the domains, in particular focusing on unidirectionally propagating waves we found in our system.

**Figure 1:**
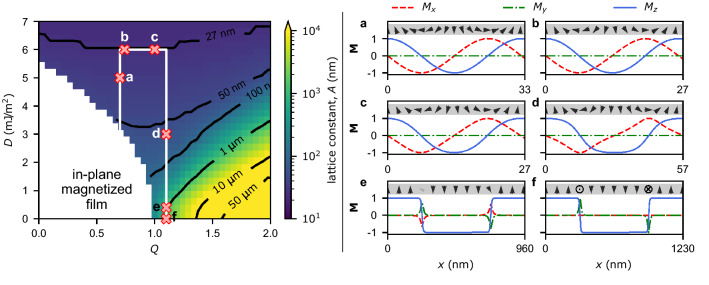
(Left panel) Color map and isolines representing the lattice constant (*A*) of periodic magnetic textures as a function of DMI (*D*) and PMA (*Q*) values. The presented simulations are done with a *Q* step of 0.05 and a *D* step of 0.25 ${\mathrm mJ/m^2}$. The white color denotes the in-plane phase in the remanence. The white line marks the trajectory in the (*D*, *Q*)-space for which the dynamics is studied. The red “X” symbols indicated combinations of (*D*, *Q*) for which special emphasis in the analysis is given. (Right panel) Magnetic configurations for the points marked in (left panel) as (**a–f**), i.e., (**a**) $Q=0.7$, $D=5$ ${\mathrm mJ/m^2}$, $A=33\,{\mathrm nm}$, (**b**) $Q=0.74$, $D=6\,{\mathrm mJ/m^2}$, $A=27\,{\mathrm nm}$, (**c**) $Q=1.0$, $D=6\,{\mathrm mJ/m^2}$, $A=27\,{\mathrm nm}$, (**d**) $Q=1.1$, $D=3\,{\mathrm mJ/m^2}$, $A=57\,{\mathrm nm}$, (**e**) $Q=1.1$, $D=0.4\,{\mathrm mJ/m^2}$, $A=960\,{\mathrm nm}$, and (**f**) $Q=1.1$, $D=0$, $A=1230\,{\mathrm nm}$. The red, green, and blue lines correspond to $M_x$, $M_y$, and $M_z$ components of the reduced magnetization, respectively. For a more illustrative representation of the magnetic configurations, we have visualized them using arrows in the gray stripes on top of each plot. These arrows depict the cross-sectional view ($(x,z){\mathrm -plane}$) of the magnetic configuration.

## Results

### Static magnetic configuration in dependence on DMI and PMA

Let us consider a 2 nm thick Co film (the saturation magnetization $M_{\textrm S}=1420\,{\mathrm kA/m}$ and the exchange constant $A_{\textrm ex}=13\,{\mathrm pJ/m}$) in the remanence (at zero external magnetic field) and analyze how the magnetic configuration depends on the DMI and PMA values. We use micromagnetic simulations^53^ to determine the magnetic configuration [${\textbf{M}}=(M_x, M_y, M_z)$, where *z* is perpendicular to the film’s surface, and *x* is the direction of periodicity] corresponding to the minimum energy density of the system. Figure 1(left panel) shows the colormap with isolines depicting the lattice constant (*A*) of magnetization textures as a function of PMA (represented as *Q*) and DMI (*D*) (see “Methods” for more details). Overall, depending on the interplay between these parameters, we observe the in-plane phase (the white region in the plot), spin spiral, or a stripe domain pattern with Néel- or Bloch-type domain walls. Exemplary magnetic configurations for points marked by ‘X’ in Fig. 1(left panel) are shown in the right panel of Fig. 1. It is visible that the period varies by more than three orders of magnitude depending on *D* and *Q*. In the case of $D=0$, we obtain stripe domains with Bloch domain walls (the out-of-plane phase) for $Q \ge 1$ and the in-plane phase for $Q<1$. It shows the physical meaning of *Q* (the ratio of PMA to demagnetization energies), confirming that the out-of-plane phase is obtained for $Q > 1$ in the limit of thickness approaching 0. The periods are relatively large, $A=250\,{\mathrm nm}$ for $Q\approx 1$, $A=1230\,{\mathrm nm}$ for $Q=1.1$, and tens of micrometers for $Q\gtrsim 1.4$.

**Figure 2:**
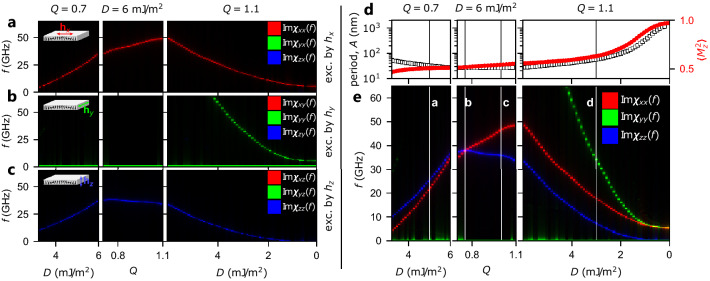
Resonance spectrum in dependence on *D* and *Q* calculated separately for the excitation by microwave field polarized along (**a**) the *x*-axis, (**b**) the *y*-axis and, (**c**) the *z*-axis (see graphical representation in top left corners of each subplot). The calculations are done along the path marked in Fig. 1. The intensity of red, green, and blue colors correspond to the intensities of the space-averaged dynamic components of the dynamical magnetization $|\langle m_x \rangle (f)|$, $|\langle m_y \rangle (f)|$, and $|\langle m_z \rangle (f)|$, respectively. Therefore, plots (**a**–**c**) represent all components of dynamical susceptibility. (**e**) Combined results from (**a**–**c**). The vertical lines labeled by bold letters *a–d* indicate the parameters selected for further simulations shown in Figs. 3 and  4 and denoted there by the same letters. (**d**) The lattice constant (the empty black squares) and space averaged square of the out-of-plane component of the magnetization $\langle M_z^2\rangle$ (the red dots) in the dependence on *D* and *Q* plotted along the path marked in Fig. 1.

Let’s examine how the magnetic configuration changes along the white path in Fig. 1(left panel). The changes of *A* and $\langle M_z^2 \rangle$ are depicted in Fig. 2d. Firstly, while DMI increases at constant *Q*, the period decreases. For the second part of the path at fixed $D=6\,{\mathrm mJ/m^2}$, the period is equal to $A=27\,{\mathrm nm}$ (see exemplary configurations in Fig. 1b,c). For $Q=1.1$ and decreasing value of *D*, *A* increases by a few orders of magnitude, and since $\langle M_z^2 \rangle$ increases, the up and down domains become distinguishable (compare Fig. 1d–f). Finally, with decreasing *D*, classical stripe domain patterns are established, with up and down domains and narrow domain walls (compared to domain size). The walls are firstly of the Néel type, followed by the Bloch type for *D* approaching 0, with some mixed Bloch–Néel configuration in between (see Fig. 1e). Overall, for most of this path, the spin spiral state with different lattice constant is the equilibrium magnetic configuration (see Fig. 1a–c).

### SW resonances in dependence on DMI and PMA

The excitation of resonances by a spatially uniform microwave field is fundamental from the application point of view. We analyze how the dynamic susceptibility^50^ ${\textrm Im} \chi _{ln}$ changes while following the trajectory marked in Fig. 1, where $l, n\in {x, y, z}$. ${\textrm Im} \chi _{ln}$ represents the response of $m_l$ component of dynamic magnetization ($\Vert {\textbf{m}}\Vert \ll M_{\textrm S}$) to the excitation by a microwave field polarized along the *n*-axis.

Separate simulations for *x*-, *y*-, and *z*-polarized microwave fields are performed (see “Methods”), and simulated spectra are shown in Fig. 2a–c, respectively. The figures utilize the RGB color model, where the response of each component of the dynamical magnetization corresponds to one of the primary colors that are additive. Namely, red, green, and blue correspond to the space-averaged response of $m_x$, $m_y$, and $m_z$, respectively. These three plots are combined in Fig. 2d. We can see that the *x*-polarized microwave field excites only one band, which has red color. Similarly, the *z*-polarized microwave field excites only one blue band, whereas, the *y*-polarized field excites two green bands. What is important from the application point of view (e.g., in sensing) in an ideally aligned magnetization pattern, using linearly polarized microwaves we can selectively excite different resonant modes. The explanation of this selectivity of excitation will be provided in the subsequent subsection.

Let us analyze how the frequencies of the presented bands changes with *D* and *Q*. For constant $Q=0.7$, while the DMI increases, the frequencies of the blue and red bands increase since the lattice constant *A* decreases. The horizontal green low-frequency band, independent of *Q* and *D* corresponds to the Goldstone mode^39^. The origin of the green color of this band will be discussed later in the paper when analyzing the dispersion relations for waves propagating along the *x* axis. For constant $D=6\,{\mathrm mJ/m^2}$ and *Q* increasing from 0.7 up to 1.1, we can observe that the blue and red bands swap order, i.e., the frequencies of the blue band decreases, whereas the frequencies of the red band grows. It leads to the crossing of the red and blue bands at $D=6\,{\mathrm mJ/m^2}$ and $Q=0.74$. Interestingly, there is no signature of the interaction between these modes, since they degenerate. A significant change of the frequencies of the blue and red bands is surprising since the lattice constant does not change, $A=27\,{\mathrm nm}$. The only visible difference occurring in the spin texture for this segment is a slight increase of the value of $\langle M_z^2 \rangle$ due to the increase of PMA strength. We will explain the different monotonicity of frequency changes of the red and blue bands in the next subsection. Finally, for the last segment where *D* decreases from $6\,{\mathrm mJ/m^2}$ down to zero at $Q=1.1$, we observe that *A* and $\langle M_z^2 \rangle$ increase (cf. in Fig. 2d). It corresponds to the gradual transition of the spin spiral into a stripe domain pattern with narrow domain walls separating flat up and down domains. As expected, we observe that the frequencies of the resonant modes decrease. Moreover, at $D \approx 4\,{\mathrm mJ/m^2}$, we notice the presence of a new green band, whose frequencies also decrease with a decrease of *D* and an increase of the lattice constant. Finally, the frequency of the blue band drops to zero as *D* approaches zero and the frequencies of the red and the higher green bands become very close to each other.

**Figure 3:**
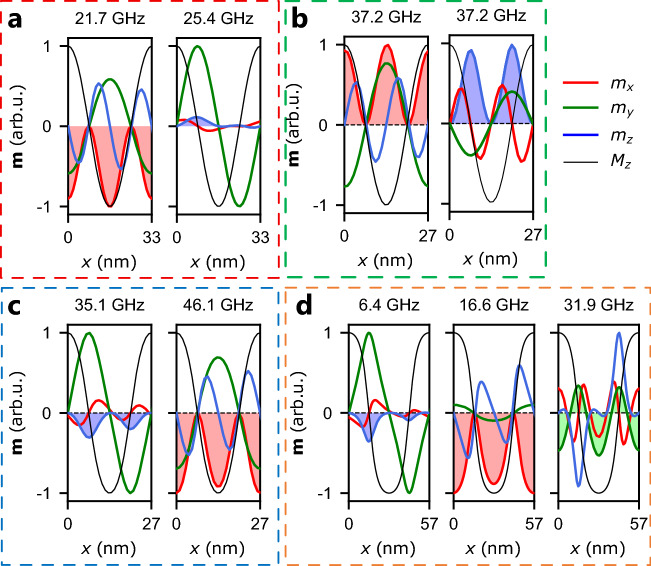
Resonance mode profiles for the same parameters (**a**–**d**) as the magnetic configurations plotted in Fig. 1a–d with kept numeration of the subplots. The thick red, green and blue lines indicate the space dependencies of the dynamic components of magnetization $m_x$, $m_y$, and $m_z$, respectively. The narrow black lines indicate the behavior of the static $M_z$-component of magnetization and serve as point of reference. The color-fill indicates the component *m* not averaging to 0. Note, that since $M_z \gg m_x, m_y, m_z$, the curves representing $M_{z}$ are rescaled to the amplitude of $\sqrt{m_x^2+m_y^2+m_z^2}$.

#### Resonance mode profiles

To understand better this spectrum, we analyze the mode profiles for four combinations of (*D*, *Q*) indicated in Fig. 2 by the vertical lines. The points *a*–*d*, marked by ‘X’ in the left panel of Fig. 1 are shown in Fig. 3a–d, respectively. We will call the modes by a color (red, green, and blue) according to the color of the band in Fig. 2 they originate from. The profiles of the red and blue modes for $Q=0.7$ and $D=5\,{\mathrm mJ/m^2}$ are shown in Fig. 3a. For the red mode at $f=21.7\,{\mathrm GHz}$, $m_x$, and $m_y$ have a twice greater period than $m_z$, and the amplitude of all three dynamical components are comparable. The $m_x$ component oscillates in-phase; therefore, it does not average to zero, whereas the space average of $m_y$ and $m_z$ is equal to zero. It explains the red color of this band in Fig. 2. The amplitudes are concentrated only in regions where $M_z \ne 0$. In the case of the blue mode ($f=25.4\,{\mathrm GHz}$), the $m_y$ amplitude is much higher than of the other components, and it is concentrated at the regions where $M_z$ is close to zero. However, it averages to zero. By contrast, the much smaller amplitude of the $m_z$ component (the blue line) does not average to zero since it does not cross zero, whereas $\langle m_x \rangle =0$. This explains the blue color of the band. It may also suggest a weaker efficiency of the excitation of the blue mode than the red mode. Furthermore, the $m_x$ component changes with doubled spatial frequency of $m_y$ and $m_z$.

Different monotonicity of frequency changes of red and blue modes and their crossing for $D=6$ mJ/m$^2$ can be explained by analyzing the resonant mode profiles. As described earlier, the red mode has its amplitude concentrated near the regions of the texture, where $M_z \ne 0$ while the blue mode is around areas with $M_z = 0$. Thus, as the contribution of PMA increases, the $\langle M_z^2 \rangle$ increases, and the spin spiral slowly transforms towards a stripe domain pattern, i.e., the regions with $|M_z| \approx 1$ flatten out. As the value of PMA increases, the energy of oscillation of deviations from the perpendicular direction increases, which explains the increase in the frequency of the red band when *Q* increases and the value of the lattice constant is maintained. In contrast, the energy of the domain-wall localized blue mode decreases as a result of the increase of the PMA trying to direct magnetic moments in a perpendicular direction. Consequently, the increase of *Q* and the flattening of magnetic domains leads to a scenario typical of stripe domains: modes located in domain walls have lower frequencies than modes located in magnetic domains^7^.

For the mode crossing at $Q=0.74$ and $D=6\,{\mathrm mJ/m^2}$ shown in Fig. 3b, we had to prepare separate simulations for the microwave field polarized along either the primary directions. It allowed us to separately visualize two degenerated modes at the same frequency. It is visible that for the first mode, the amplitude of $m_x$ component does not average to zero, whereas, for the second mode, the amplitude of $m_z$ does not average to zero. This is an important result because it shows that even for degenerated modes, we can decide which mode will be excited by choosing the polarization of the microwave field. Moreover, this clearly confirms that the red and blue modes do not interact with each other. Interestingly, in the simulation where we assumed the linear polarization of the microwave field along the diagonal axis [1, 1, 1], only the red mode was visible, which confirms that it is much easier to be excited.

For the third set of parameters ($Q=1$ and $D=6\,{\mathrm mJ/m^2}$) shown in Fig. 3c, the blue mode swaps order with the red mode. However, the mode profiles do not change significantly with respect to Fig. 3a. Finally, for the fourth set of parameters depicted in Fig. 3d, we observe similar profiles of the first two modes as in the previous set of parameters. This spatial distribution is slightly modified due to the flattening of the regions where $|M_z|\approx 1$, see Fig. 3d. Moreover, a new green mode appears in the considered frequency range. It is a mode with the amplitude concentrated within the up and down domains and quantized across the domain widths. Therefore, its frequency is much greater than 65 GHz for smaller lattice constants. In the case of this mode, only $m_y$ does not average to zero, which explains its green color in the spectrum.

The mode profiles for the blue band are characterized by large amplitude oscillation of $m_y$ and much weaker oscillations of $m_x$ and $m_z$ (cf. Fig. 3, the only exception is visible for the point of crossing visible in Fig. 3c). Therefore, one might see the resemblance of dynamic magnetization distribution corresponding to the blue band and Bloch-type domain walls. This is particularly intriguing since the frequencies of the blue band for *D* approaching 0 drop down to $f=0$, and it coincides with the emergence of Bloch-type domain walls with magnetization rotated towards *y*-axis (with $M_y= \pm 1$) separating up and down domains. It may suggest a close connection of this mode with the Bloch-type domain wall; however, this topic requires further study.

**Figure 4:**
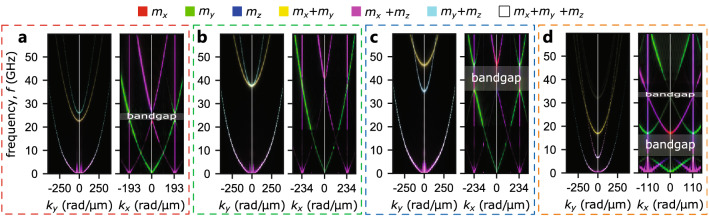
Dispersion relations for SWs propagation along the *y*-axis ($k_y$, left panels) and along the *x*-axis ($k_x$, right panels) for the selected sets of parameters used in Fig. 3 and kept numeration of subplots. The RGB color model is utilized to depict the oscillation’s amplitudes of $|m_x|$ (red color), $|m_y|$ (green color), and $|m_z|$ (blue color). Thus, cyan, magenta, and yellow (as additive mixtures of RGB components) depict dominating oscillations of $m_y$ and $m_z$, $m_x$ and $m_z$, $m_x$ and $m_y$, respectively. White color indicates oscillations of all three components. The gray horizontal bars denote positions of bandgaps.

**Figure 5:**
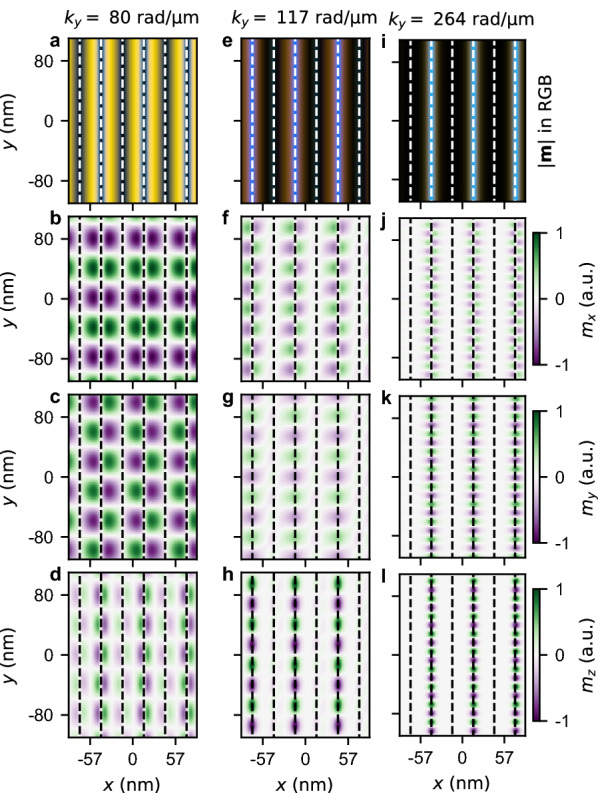
Visualization of the third mode profile of SWs for $D=3$ mJ/m$^2$ and $Q=1.1$ (point (d) in Fig. 1) and frequency 20 GHz for (**a**–**d**) $k_y= 80$ rad/${\upmu }$m, (**e**–**h**) $k_y= 117$ rad/${\upmu }$m, and (**i**–**l**) $k_y= 264$ rad/${\upmu }$m. (**a**,**f**,**i**) The representation of the absolute value of the amplitude of SWs $\mathbf {|m|}$ in the RGB color scheme. (**b**,**f**,**j**), (**c**,**g**,**k**), and (**d**,**h**,**l**) the distribution of the amplitude of the real part of $m_x$, $m_y$, and $m_z$, respectively. The dashed vertical lines represents the positions for which $M_z=0$ (domain walls).

### Dispersion relations

Let us focus on the propagation of SWs in these textures for two basic propagation directions, i.e., along the *x*- and *y*-axis. Noteworthy, the presented before mode profiles can be understood as SWs with wavevector $k=0$. The dispersion relation dependencies on $k_x$ and $k_y$ displayed in Fig. 4 are computed for the same sets of parameters as the mode profiles shown in Fig. 3. Here, again, we utilize the same RGB color model to depict oscillations of the $|m_x|$, $|m_y|$ , and $|m_z|$. The intensity of the absolute value of each component is related to the amplitude of the corresponding primary color (see “Methods” for details).

#### Propagation along the *y*-axis (along domains)

For the propagation of SWs along the *y*-axis, we see that the dispersion relation is reciprocal similarly as in weak stripe domain patterns^12,28^. We see that the hue of the three lowest bands differs, and we can distinguish magenta, yellow and cyan bands. For higher frequencies, the magenta band turns pale cyan or white, and the yellow and cyan bands swap order (cf. Fig. 4a–c) similar to the red and blue bands in Fig. 2. The hue indicates the type of the mode. The yellow band result from the dominating oscillations of the $m_x$ and $m_y$ components of magnetization and, therefore, it is mostly concentrated in the regions with $|M_z|$ approaching 1. The cyan band is related to the oscillation of $m_y$ and $m_z$, thus, it is mostly concentrated in the regions with $|M_x|$ approaching 1. Finally, the lowest band turns magenta for low frequencies (oscillations of $m_x$ and $m_z$). Since for $k=0$ the frequency is equal to zero, the origin of this mode can be understood as the domain wall flexure oscillation. One can think about this mode as a counterpart of the Winter’s magnons^5^ for the magnetic moments rotating in the $(x,z){\mathrm -plane}$ similarly as a Néel-type domain wall. Furthermore, comparing Figs. 2 and  4 we can conclude that the two lowest bands for $D<1$ mJ/m$^2$ merge at $f=0$ and represent an oscillation of Bloch-type domain wall, i.e., classical Winter’s magnon^5^.

To better understand the nature of these three bands, let us analyze the mode profiles of SWs with $k_y>0$ at frequency 20 GHz for the system with $D=3$ mJ/m$^2$ and $Q=1.1$, see Fig. 5. The first mode profile represent SWs with the amplitude concentrated in regions with $M_z \ne 0$, cf. Fig. 5a–d. We can see that the amplitude of this mode in the RGB representation turns yellow. It occurs because in the regions where $M_z \ne 0$, the amplitude of $m_x$ and $m_y$ is substantially larger than the amplitude of $m_z$. It also explains the yellow color of the band in the dispersion relation. The second and the third modes, shown in Figs. 5e–h and 5i–l, respectively, depicts SWs concentrated in regions around $M_z=0$. For the sake of simplicity and consistency with stripe up and down domains, let us call these regions domain walls. We can observe that the amplitudes of these modes are localized in every second domain wall (different for the second and third modes). It is an analogous observation as in the case of weak stripe domain patterns^2,14,28^, where the unidirectionality was found for thicker films without DMI and caused by the complex corkscrew type internal structure of domain walls and dipole interactions. Finally, in the RGB representation of the SW amplitude, we can see that these unidirectional modes are blue-dyed. It agrees with the color of the cyan band in the dispersion relation.

#### Propagation along the *x*-axis (across domains)

For the propagation along the *x*-direction (right panels in Fig. 4a–d), we observe that the dispersion is periodic, as in the case of magnonic crystals. However, unlike for weak stripe domains^12,14^, here, all the bands change monotonically within one Brillouin zone. We can distinguish two colors of the bands, green (oscillations of $m_y$) and magenta (oscillations of $m_x$ and $m_z$). These bands look similar; however, the green bands are shifted by $2\pi /A$ with respect to magenta bands. The periodicity of the green and magenta bands in the reciprocal space equals $4\pi /A$, i.e., it is twice greater than the texture’s periodicity in the real space suggests. These are similar results as reported in the case of weak stripe domain patterns^12^. It is caused by the symmetry of the mode profiles. For instance, the Goldstone mode^12^ for $m_x$ and $m_z$ correspond in the spectrum to the points $(f=0, k=2\pi (2n+1)/A)$ (for $n=0,\pm 1,\pm 2,\ldots$), whereas for $m_y$ to $(f=0, k=4\pi n/A)$. As a side note, the fact that the purely green band descends to zero frequency at $k_x=0$ also explains the green color of the band for $f=0$ in the resonance spectrum shown in Fig. 2 since at $f=0$ with $k=0$ only $m_y$ oscillates. In general, all green bands are shifted along the $k_x$-axis with respect to the magenta bands. A simplified explanation of this effect on the example of a fundamental mode can be found in the supplementary materials of Ref.^35^. A similar effect occurs in the frequency domain for elliptical precession, where different temporal frequencies of oscillations of different magnetization components are present. This effect is used in parametric parallel pumping^54^, where the magnetization component aligned with the effective field oscillates with twice greater temporal frequency than the orthogonal components^55^. Here, we observe an equivalent effect in the wavevector-space where the spatial frequency is different for $m_y$ than for $m_x$ and $m_z$.

## Discussion

The results of our numerical study have many important implications. Let us first discuss the merging of the two lowest bands for $Q>1$ and low DMI values visible for both FMR spectra dependence on *D* and for dispersion relations, cf., Figs. 2 and  4, respectively. Similarly, as for the propagation along the *y*-axis, the two lowest bands for $Q=1.1$ drops to $f=0$ for *D* approaching 0. This is caused by very large separations between domain walls which, therefore, decrease the strength of the coupling between them. Moreover, the lowest two bands do not backfold at the boundary of the Brillouin zone. It further confirms our interpretation of the origin of the first two bands as magnetization texture oscillations originating from the Goldstone mode. For all the higher bands, the backfolding occurs at the boundary of the Brillouin zone; thus, we associate them with typical SW excitations. Accordingly, we can distinguish two types of bandgaps, (1) between the texture’s and SW’s bands and (2) bandgaps resulting from the interaction of SWs. Since there is no interaction between SWs and the texture oscillations, the first bandgap closes in Fig. 4b.

**Figure 6:**
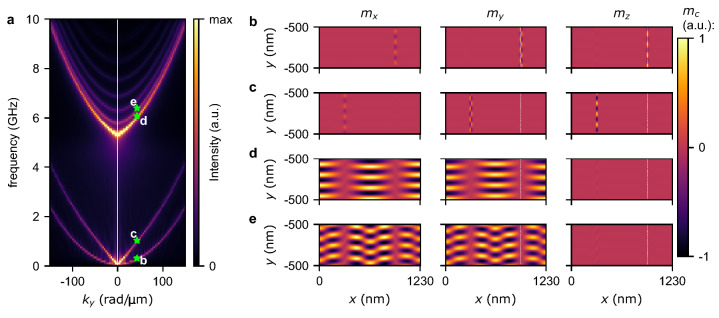
(**a**) Dispersion relations for SWs propagation along the *y*-axis for $Q=1.1$ and $D=0$. (**b**–**e**) Mode profiles calculated for $k_y=50$ rad/${\upmu }$m and subsequently the frequencies 0.3 GHz, 1.0 GHz, 6.1 GHz, and 6.4 GHz. These modes correspond to the points marked on the dispersion relation as green stars annotated by the letters b–e. Mode profiles are separately visualized for $m_x$, $m_y$, and $m_z$ components of magnetization in the left, central, and right columns, respectively. The vertical white dashed lines denote the positions of domain walls where $M_z=0$.

**Figure 7:**
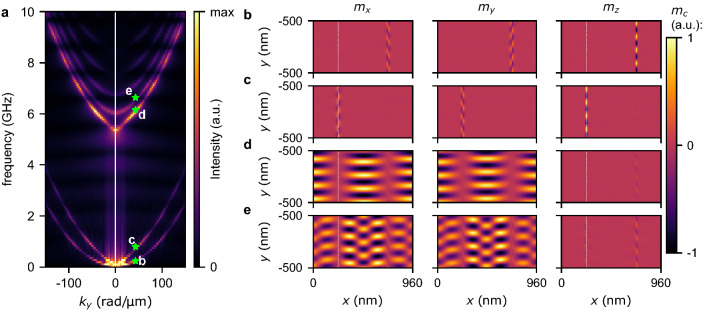
(**a**) Dispersion relations for SWs propagation along the *y*-axis for $Q=1.1$ and $D=0.4$ mJ/m$^2$. (**b**–**e**) Mode profiles calculated for $k_y=50$ rad/${\upmu }$m and subsequently the frequencies 0.2 GHz, 0.8 GHz, 6.2 GHz, and 6.6 GHz. These modes correspond to the points marked on the dispersion relation as green stars annotated by the letters b-e. Mode profiles are separately visualized for $m_x$, $m_y$, and $m_z$ components of magnetization in the left, central, and right columns, respectively. The vertical white dashed lines denote the positions of domain walls where $M_z=0$.

Let us now discuss the propagation of SWs along the *y*-axis in the range of parameters where the first two bands degenerate at $k_y=0$ and $f=0$, which corresponds to the stripe domain patterns characterized by larger lattice constants. Here, we focus on the propagation along the *y*-axis for $Q=1.1$ and two values of DMI, i.e., $D=0.4$ and $D=0$ mJ/m$^2$. These combinations of *D* and *Q* correspond to the magnetic configurations shown in Fig. 1e,f that are characterized by lattice constants being tens of times larger than for the system previously used to calculate dispersion relations. In order to reduce the required resources to run the simulation, we performed simulations for a single lattice constant with assumed periodic boundary conditions (see details in “Methods”). This assumption forced a robust period of the simulated system. Figures 6 and 7 show the dispersion relations and mode profiles for the first four bands for $D=0$ and $D=0.4$ mJ/m$^2$, respectively. These two dispersion relations are very similar to each other. It can be seen that the first two bands for $k_y=0$ are degenerated at $f=0$ and represent waves located in the domain walls. For $D=0$ the higher frequency bands representing SWs (from the third band up) are more closely packed than for $D=0.4$ mJ/m$^2$. It can be seen that a wave localized in and propagating along a domain wall have different wavelengths depending on the direction of propagation, cf. Figs. 6b,c and 7b,c (it is a similar result as for spin spirals, see Fig. 5). The third and higher bands represent successive modes localized in magnetic domains that differentiate by quantization, see Figs. 6d,e and 7d,e. At $k_y=0$, the third and fourth bands correspond to the red and green bands in the resonance spectrum, cf. Fig. 2e.

For the range of *D* values for which the resonance frequencies of the first two bands degenerate at $f=0$ (see Fig. 2), we were unable to determine dispersion relations for the propagation along the *x*-axis (the resulting images were very noisy without visible magnonic bands). This was because the magnetic configuration was slightly changing during the simulation, although the average period remained the same as in our static simulations. We interpret this result that in ultra-thin films, DMI stabilizes regular magnetic textures with a constant period similar to the dipole interactions in the case of thicker layers and multilayers^12,13^. This result seems intuitive because, looking at the dispersion relations for propagation along the *x*-axis, one can see that as DMI decreases, the frequencies of the first two bands decrease. This indicates that for a value of *D* for which the resonant frequency of the second mode (waves at $k=0$) drops to zero, the first two bands merge at $f=0$ in the whole $k_y$ range. Our interpretation indicates that the magnetic texture for this scenario is not stable enough to allow us to determine a clear dispersion relation and thus cannot be applicable as a magnonic crystal. This is an intriguing subject that requires further research in the future.

The behavior of SWs at $Q=0.74$ and $D=6\,{\mathrm mJ/m^2}$ is particularly interesting. For the propagation along the *x*-axis, there is a linear band crossing of the second and third band at $k=0$ with no sign of hybridization. At this point occurs the swap of the bands order of modes associated with SWs and texture oscillations, i.e., this is the point where the order of the cyan and yellow bands changes for the propagation along the *y*-axis and the blue and red bands swap in FMR spectrum (cf. Fig. 2). Changing the order of the bands for the set of parameters when the linear crossing of the bands occurs is a typical feature of the topological transition point^56^. It indicates a very interesting direction of further research in magnonics and the topological properties of our system will be studied in details in the future.

**Figure 8:**
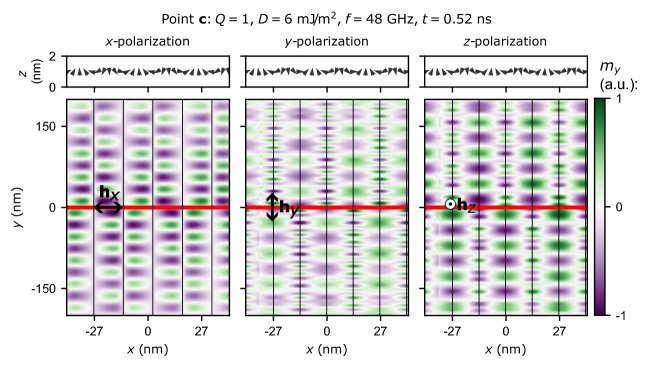
Simulations of the excitation of SWs propagating along the *y*-axis for $Q=1$ and $D=6$ mJ/m$^2$ for three different polarizations of the microwave field obtained after 0.52 ns of continuous excitation of SWs. (Upper row) Cross-sectional view of the magnetic configuration. (Bottom row) Colormaps representing $m_y$ for the SWs excited by the microwave field applied in the regions indicated by the red line for (1) the *x*-polarized microwave field (left column), (2) the *y*-polarized microwave field (central column), and (3) the *z*-polarized microwave field (right column). The vertical black lines denote the regions where $M_z=0$.

Different colors of the bands for propagation along domains (the *y*-axis) in Fig. 4 suggest the possibility of selective excitation of waves depending on the polarization of the locally applied microwave field. Accordingly, let us analyze the influence of the polarization of a locally applied microwave field on the emission of SWs propagating along the *y*-axis. Instead of studying the eigensolutions of the system as previously, let us examine the system’s response after a certain time of continuous SW emission. In our case, 0.52 ns is long enough to achieve the steady-state. SWs are excited by a locally applied microwave field in the region of a width of $w=10$ nm centered at $y=0$ indicated by the red color in Fig. 8 where the amplitude distribution of the $m_y$ component of magnetization is shown. The simulations are performed for $Q=1$ and $D=6$ mJ/m$^2$ and for three different polarizations of the microwave field. Namely, we use the microwave field at the frequency $f_0$ with the distribution described by the function ${\textbf{h}}_{c}(t, y) = h_0 {\textrm sin}(2\pi f_0 t){\textrm rect}(y/w) \Sigma _j \delta _{cj} {\textbf{e}}_j$, where ${\textrm rect}$ is the rectangular function, $\mu _0 h_0= 10$ ${\upmu }$T, $c\in \{x,y,z\}$ denotes the component of magnetization, $\delta _{cj}$ is the Kronecker delta, and ${\textbf{e}}_c$ is the *c*-th unit vector. The results for the microwave field polarized along the *x*-, *y*-, and *z*-axis and shown in the left, central, and right columns in Fig. 8.

Analysis of Fig. 8 reveals that the *x*-polarized microwave field excites the mode with amplitude of $m_y$ concentrated in regions where $M_z \ne 0$. This mode corresponds to the yellow band in Fig. 4. On the other hand, microwave field polarized along the *y*- or *z*-axis excites both modes with amplitude of $m_y$ concentrated in regions where $M_z=0$. The unidirectionality of the propagation is well visible for these two bands. For each domain wall (the region where $M_z=0$), we have different wavelengths for waves propagating along the $+y$ and $-y$ directions. Moreover, this picture changes in every second domain wall. Shorter wavelengths alternately rotate in successive domain walls along the $-y$ and $+y$ directions, while longer wavelengths propagate along the $+y$ and $-y$ directions. This observation is in agreement with the mode profiles shown for positive $k_y$ in Fig. 5. Similar effect was also observed for weak stripe domains^2,14,28^. Furthermore, as shown in Fig. 4c,d, the second and third bands (bulk- and DW-type) cross without hybridization because they have different origins and do not interact with each other. It indicates that there is no energy transfer between them. It is important from an application point of view and suggests that transmission of information using these two modes without crosstalk between these two channels is possible.

Summarizing, we have observed that the resonance frequencies change significantly due to DMI- and PMA-induced changes in the magnetic configuration. We decided to analyze SW dynamics also in complete spin spirals, which required to analyze systems with DMI values up to 7 m*J/m*^2^, although the experimentally obtained samples show DMI values not exceeding 2.5 m*J/m*$^2$^57^, Nevertheless, even though some of the calculations were performed for large DMI values, most of the obtained effects should also occur for a smaller range of DMI values. We have observed and explained the selectivity of excitation of the resonance modes by spatially uniform microwave fields of various polarizations. We obtained dispersion relations characteristic for one-dimensional magnonic crystals with well-pronounced bandgaps and lattice constants unattainable by conventional methods. Our analysis revealed that the first two bands are associated with magnetization texture oscillations. In contrast, the remaining bands are associated with SWs, which explains no interaction between the modes and allows closing the first bandgap for a specific combination of DMI and PMA values. When the band gap is reopened, the bands between which the gap was formed change in order. This suggests the possibility of existence of interesting topological effects (e.g. topological transition points) related to SWs in spin spirals and stripe domain patterns. Our results indicate selective emission of different modes propagating along the domains. Particularly interesting are unidirectionally propagating SWs along regions with zero out-of-plane magnetization components. By analyzing the dynamics of SWs, we also pointed out the role of DMI in stabilizing magnetic textures in ultrathin layers with a regular robust period. Overall, our study reveals fundamental properties of stripe domains and spin spirals, and suggests the potential of using these structures in sensing applications, as a medium for information transmission, or as a magnonic crystal.

## Methods

We perform micromagnetic simulations by means of mumax3^53^ solving full Landau–Lifshitz equation using time-domain finite difference method to study the changes of the equilibrium magnetization configuration and magnetization dynamics in 2 nm thick Co film. In all the calculations we have used solver using Fehlberg method since it provided much better stability of the results than the default solver of mumax3. In the simulations, we use the following magnetic parameters: the saturation magnetization $M_{\textrm S}=1420\,{\mathrm kA/m}$ and the exchange constant $A_{\textrm ex}=13\,{\mathrm pJ/m}$, and damping constant $\alpha =0.008$ and varied values of interfacial Dzyaloshinskii–Moriya interaction strength (DMI, *D*) and quality factor $Q=K_{\textrm PMA}/($½$\mu _0 M_{\textrm S}^2)$ where $K_{\textrm PMA}$ is the perpendicular magnetocrystalline anisotropy (PMA) constant. The simulated system is discretized by unit cells of size $c_x \times c_y \times c_z$ with $c_x=c_y=1$ nm and $c_z=2$ nm. We assume periodic boundary conditions along the *x* and *y* axes. In subsequent subsections, details of simulations used to prepare Figs. 1, 2, 3, 4 and 5 from the main part of the paper are described.

### Simulations of static properties of the system

The aim of the static simulations is to find the equilibrium magnetic configuration and its lattice constant *A* (if the resulting texture is periodic) for each set of (*D*, *Q*). We perform the simulations for a system of size $10 A \times c_y \times c_z$. The algorithm for these simulations is as follows. For each combination (*D*, *Q*), we assume the initial magnetic configuration with the following dependence of the out-of-plane component of the reduced magnetization $M_z={\textrm sin}(2\pi x / A)$, we relax it, and we analyze changes of the energy density for different values of *A*. We then choose the magnetic configuration corresponding to the minimum energy density and use it to prepare Fig. 1 and in all the further simulations as the initial magnetic configuration.

### Simulations of the resonance spectrum

To calculate the resonance spectrum shown in Fig. 2, we use the system of size $40A \times 8c_y \times c_z$, and for each set of parameters, we perform three separate simulations for three different linear polarizations of the microwave field: (1) *x*-polarized microwave field ${\textbf{h}}_x = [h_0 {\textrm sinc}(2\pi f_{\textrm cut} (t-t_0)), 0, 0]$; (2) *y*-polarized microwave field ${\textbf{h}}_y = [0, h_0 {\textrm sinc}(2\pi f_{\textrm cut} (t-t_0)), 0]$; (3) *z*-polarized microwave field ${\textbf{h}}_z = [0, 0, h_0 {\textrm sinc}(2\pi f_{\textrm cut} (t-t_0))]$, where $\mu _0 h_0=10$ ${\upmu }$T, $f_{\textrm cut}=65$ GHz is the cut-off frequency, and $t_0=8/f_{\textrm cut}$ is the time delay. The results of simulations are sampled with the sampling interval $t_{\textrm sampling}=1/(2.2 f_{\textrm cut})$. Finally, we store a number of samples providing the spectral resolution down to 200 MHz.

As a second step, for each simulation and for each component of magnetization, we calculate fast Fourier transform (FFT) over time of the space averaged magnetization,

1 $$\begin{aligned} {\widetilde{m}}_{c,p}(f)=\frac{2}{N} \Big | {\mathscr {F}}_t \left[ \langle m_{c,p}(t; x, y, z) \rangle _{x,y,z} \right] \Big |, \end{aligned}$$

where ${\mathscr {F}}_t$ denotes FFT operation over time implemented in NumPy^58,59^, *N* is the number of samples processed, $c\in \{x,y,z\}$ denotes component of magnetization, and $p\in \{x,y,z\}$ denotes the polarization of the applied microwave field. Since, to excite SWs we use microwave field with time dependence described by ${\textbf{h}}_{p} \propto h_0{\textrm sinc}(2\pi f_{\textrm cut})$, its amplitude in the frequency space is $h_0$ if $f<f_{\textrm cut}$ and 0 if $f > f_{\textrm cut}$. Therefore, normalized ${\widetilde{m}}_{c,p}(f)$ can be understood as a dynamical susceptibility ${\textrm Im}\chi _{c,p}(f)$ describing the dynamical response of *c*-th component on the excitation by microwave field polarized along the *p*-axis. For presentation, each spectrum ${\textrm Im}\chi _{c,p}(f)$ for a given set of parameters (*Q*, *D*) is normalized to 1. Finally, we utilize the RGB color model where each component of dynamical magnetization corresponds to one of the primary colors that are additive, i.e., the red, green, and blue color denotes the amplitude of ${\textrm Im}\chi _{x,p}(f)$, ${\textrm Im}\chi _{y,p}(f)$, and ${\textrm Im}\chi _{z,p}(f)$, respectively.

### Resonance mode profiles

To obtain the mode profiles shown in Fig. 3, we have performed simulations using a spatially uniform microwave field linearly polarized along the axis [1, 1, 1]:

2 $$\begin{aligned} \mu _0 {\textbf{h}} = \mu _0 h_0 {\textrm sinc}(2\pi f_{\textrm cut} (t-t_0)) [1, 1, 1], \end{aligned}$$

At the crossing point ($Q = 0.74$ and $D = 6$ mJ/m$^2$), in order to plot the profiles of degenerate modes separately, two independent simulations were performed for microwave fields polarized along the *x*-axis ($\mu _0 {\textbf{h}} \propto [1,0,0]$) and along the *z*-axis ($\mu _0 {\textbf{h}} \propto [0,0,1]$).

To get the mode profiles, we calculate the pointwise FFT over time of each component of magnetization:

3 $$\begin{aligned} {\widetilde{m}}_c(f;x,y,z)=\frac{2}{N}{\mathscr {F}}_t \left[ m_c(t; x, y, z) \right] . \end{aligned}$$

Subsequently, we visualize the real part of ${\widetilde{m}}_c$ for subsequent resonance frequencies $f_{\textrm resonance}$ as the mode profiles, i.e., ${\textrm Real}[{\widetilde{m}}_i(f_{\textrm resonance};x,y,z)]$.

### Dispersion relations

To simulate the dispersion relation shown in Fig. 4, we use the system of size $40A \times 1000\,{\mathrm nm} \times 2\,{\mathrm nm}$. In order to determine the dispersion relation of SWs propagating along the *y*-axis with proper resolution, the modeled system needs to be sufficiently long. Namely, the length of the system along the *y* axis determines the resolution of $k_y$ (minimal resolved $k_y$, whereas and the number of unit cells defines the maximal resolved $k_y$ after FFT. In the case of determining the spectrum, that is, the response of the system for $k_y=0$, it was sufficient to model a much shorter system (8 nm) with periodic boundary conditions. To determine the dispersion relation of SWs propagating along the *x*-axis, we excite the magnetization dynamics with a microwave field linearly polarized along the axis [1, 1, 1]:

4 $$\begin{aligned} \mu _0 {\textbf{h}} = \mu _0 h_0 {\textrm sinc}(2\pi f_{\textrm cut} (t-t_0)) {\textrm sinc}(k_{\textrm cut}x) [1, 1, 1], \end{aligned}$$

where $\mu _0 h_0=10$ ${\upmu }$T and $k_{\textrm cut}=2\pi /(20\,{\mathrm nm})$ is the cut-off wavevector. We use the same values of $f_{\textrm cut}$ and $t_0$ as to calculate the resonance spectrum.

Subsequently, for each component of magnetization and each value of *y* (different slices of the modeled system corresponding to different values of *y*), we calculate 2-dimensional-FFT^58,59^ over the *x*-coordinate and time *t*, denoted by the operator ${\mathscr {F}}_{t,x}$. This allows us to determine the three-dimensional complex matrix ${\tilde{m}}_c(y; f, k_x)={\mathscr {F}}_{t,x} \left[ m_c (t; x, y) \right]$ depending on the *y*-coordinate, frequency *f*, and wavevector $k_x$. Next, we average over the *y*-coordinate the absolute value of the matrix ${\tilde{m}}_c$: $S_c(f, k_x)=\langle |\tilde{m_c}(y; f, k_x)|\rangle _y$. The resulting $S_c(f, k_x)$ matrices consist of positive real numbers and represent the dispersion relation computed for all magnetization components. Finally, we plot dispersion again utilizing the RGB color model in such a way that the amplitudes $S_x$, $S_y$, and $S_z$ correspond to red, green, and blue colors, respectively.

Similarly, to simulate the dispersion relation for propagation along the *y*-axis, we use the following microwave field:

5 $$\begin{aligned} \mu _0 {\textbf{h}}_y(t; y) = \mu _0 h_0 {\textrm sinc}(2\pi f_{\textrm cut} (t-t_0)) {\textrm sinc}(k_{\textrm cut}y) [1, 1, 1]. \end{aligned}$$

Subsequently, for each value of *x* and for each component of magnetization, we calculate 2-dimensional-FFT over the *y*-coordinate and time *t* to get ${\tilde{m}}_c(x; f, k_y)$. Finally, we average these matrices over the *x*-coordinate to get $S_c(f, k_y)=\langle |{\tilde{m}}_c(x; f, k_y)|\rangle _x$ and present this result using the RGB color model.

The dispersion relations determined in Figs. 6a and  7a were determined for systems with lattice constants equal to 960 nm and 1230 nm (which are significantly longer periods than for systems with dispersion relations shown in Fig. 4). Due to lower frequencies of the resonant modes, in these simulations, we used a lover value of the cut-off frequency, $f_{\textrm cut}=10$ GHz. For computational reasons, these calculations were performed for a single lattice constant in the *x*-direction and a length of 4 ${\upmu }$*m* with periodic boundary conditions applied (64 repetitions of the system along the *x*-axis and 16 repetitions along the *y*-axis). The dispersion relations themselves shown in Figs. 6a and 7a represent $S(f, k_y) = S_x(f, k_y)+S_y(f, k_y)+S_z(f, k_y)$.

In order to determine the mode profiles of a propagating wave with $f=f_0$ and $k_y=k_{y0}$, e.g., shown in Figs. 5, 6b–e, and 7b–e, the following procedure is performed. First, we determine ${\tilde{m}}_{c,f_0,k_{y,0}}(x; k_y) = {\tilde{m}}_c(f=f_0, x; k_y)\delta (k_y - k_{y0})$, where $\delta (k_y - k_{y0})$ is the Dirac delta. Subsequently, we compute the inverse FFT of the result, $\tilde{{\tilde{m}}}_{c,f_0,k_{y0}} = {\mathscr {F}}^{-1}_{k_y}\left[ {\tilde{m}}_{c,f_0}(x, k_y) \right]$, and obtain the mode profile of a wave propagating with the frequency $f_0$ and the wavevector $k_{y0}$.

## Data Availability

The data that support the findings of this study are available from the corresponding author upon reasonable request.
